# A reduction in the pupil’s response to affective sounds in psychopathy and related personality traits

**DOI:** 10.14814/phy2.15235

**Published:** 2022-03-21

**Authors:** Nicola S. Gray, Aimee McKinnon, Robert J. Snowden

**Affiliations:** ^1^ Swansea University & Swansea Bay University Health Board Swansea UK; ^2^ Swansea University Swansea UK; ^3^ Cardiff University Cardiff UK

**Keywords:** Cloninger TCI, emotion, psychobiological model of personality, psychopathy, pupillometry, SRP‐4, triarchic model of psychopathy, TriPM

## Abstract

The pupil of the eye dilates in response to affective information, even if that information is not visual. We used this affective modulation of the pupil to examine the hypothesis that individuals with high traits of psychopathy have an insensitivity to emotional stimuli. We also examined general personality traits related to psychopathy. A sample of 120 healthy young men had their pupils monitored while they listened to sound clips that conveyed either neutral emotion (e.g., rain), negative emotion (e.g., a person screaming) or positive emotion (e.g., people laughing). Psychopathy and general personality traits were measured via self‐report questionnaire. As expected, both the positive and negative emotional sounds produced greater dilation in the pupil size than neutral sounds. This affective modulation of the pupil was found to be reduced for the negative sounds for people high on the “callous/affective” components of psychopathy (the Affective facet of the SRP‐4 and the Meanness scale of the TriPM) and the general personality traits of Reward Dependence and Cooperativeness. The results indicate that these callous traits of psychopathy and general personality may be underpinned by a reduction in the ability to effectively process or monitor emotional stimuli.

## Graphical abstract

The eye's pupil reacts if we hear threatening sounds. This effects was found to diminish in those with psychopathic traits, especially the traits related to poor emotional processing.

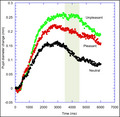

## 1 INTRODUCTION

An inability to process the affective aspects of the world is thought to lie at the heart of the psychopathic condition (Herpertz & Sass, 2000). However, data supporting this idea are mixed, and no single paradigm has produced consistent support for this theoretical position. For instance, Olderbak et al. (2018) reviewed previous meta‐analyses of emotional processing in psychopathy and conclude that “*Overall*, *the extent to which deficits are emotion specific is unclear*, *as well as which emotions are affected*, *and the magnitude of deficits*.” (pp. 296). Indeed, their own study of the issue that examined the processing of facial expressions in a large (>300) sample of male offenders failed to find any emotional deficits related to psychopathy once the possible confounding effects of intelligence were taken into account.

One possible reason for this mixed evidence is that psychopathy may not be a unitary construct. Current theories of psychopathy, alongside measurement instruments of the psychopathic construct which evolved from these theoretical positions, divide the disorder into subscales (Lilienfeld, 2018). The clinically administered Psychopathy Checklist‐Revised (PCL‐R: Hare, 2003), the most influential attempt to define and measure psychopathy, has been traditionally split into two factors: Interpersonal/affective (Factor 1) and Lifestyle/antisocial (Factor 2). However, these factors were further divided to produce a four‐facet model with Factor 1 being split to produce facets of Interpersonal (Facet 1: a tendency to manipulate others for selfish reasons) and Affective (Facet 2: a lack of concern for others), and the original Factor 2 was split into the facets of Lifestyle (Facet 3: a self‐indulgent and reckless lifestyle) and Antisocial (Facet 4: a dissocial temperament and rule‐breaking). There is strong support based on factor‐analysis for this model (Hare & Neumann, 2008). The Self‐Report Psychopathy Scale 4th Edition (SRP‐4; Paulhus et al., 2016) is a self‐report scale that has the same factor structure as the PCL‐R (Paulhus et al., 2016).

Patrick et al. proposed the Triarchic model of psychopathy (TriPM: Patrick et al., 2009). They noted that Cleckley’s (1976) conceptualized psychopaths as being both fearless/bold (showing charm, lack of anxiety or internalizing problems) and yet lacking in behavioral constraints or impulsivity (termed disinhibition). However, the PCL‐R conceptualization emphasizes meanness (deficits in empathy, cruelty or indifference to others) and disinhibition. They proposed that psychopathy is underpinned by these three phenotypic constructs: boldness (confidence and social assertiveness, emotional resilience, low anxiousness), meanness (callousness, cruelty, predatory aggression), and disinhibition (impulsiveness, irresponsibility, ager/hostility). There is also strong support for this three‐factor model, with clear demonstrations that the different scales are associated with different emotional vulnerabilities and behaviors (Patrick & Drislane, 2015; Weidacker et al., 2017)—though see Roy et al. (2021) and Pink et al. (2022).

If an emotional deficit is at the heart of psychopathy to which factor(s) does this deficit belong? The answer to this question may depend upon the task used to measure it. Perhaps the most replicated finding is that psychopathic individuals show a reduction in the fear potentiated startle response (Patrick et al., 1993)^1^. The normal fear potentiated startle response is thought to arise due to the brain's defensive motivational system, which is activated by the fear‐inducing stimulus and potentiates the body's startle to a loud noise. Vaidyanathan et al. (2011) replicated the finding that psychopaths have a reduced fear potentiated startle response but found this was only for individuals with high Factor 1 scores. Vanman et al. (2003) also found a reduced fear potentiated startle associated with Factor 1 but found that the variance unique to Factor 2 is associated with *greater* fear potentiated startle. If this is a reliable finding, it demonstrates the importance of considering each of the dimensions of psychopathy in isolation (as well as in combination) as these opposite effects may not be apparent when evaluating the global scale, as they will act to cancel each other out (Lilienfeld, 2018).

The reduction in fear potentiated startle response has also been examined via the triarchic model of psychopathy in a mixed‐gender community sample. Esteller et al. (2016) found that the fear potentiated startle response reduction was only for the Boldness scale of the TriPM. Further, this reduction was only found for visual stimuli that depicted threats, and not for those that depicted another negative valence (e.g., mutilations).

The fear potentiated startle response is thought to occur due to activity in the defensive motivational system. However, there are many other measures of emotional processing that may not involve this defensive motivational system. For instance, the pupil is known to dilate to stimuli that have strong affective content (Bradley et al., 2008; Snowden et al., 2016) and so could be used as an index of the extent of processing of the emotional content of a stimulus. The affective modulation of the pupil is believed to occur through the sympathetic nervous system and is thought to reflect a summative index of cognitive‐affective brain activity triggered by a stimulus (Siegle et al., 2003). Hence, indexing emotional processing via the pupillary response may provide a complementary method to explore emotional processes to the modulation of startle responses, and it seems feasible that the technique might produce different result due to the differences in the brain areas involved in the two phenomena.

Studies of the affective modulation of the pupil in psychopathic individuals are limited. Burley et al. (2019) tested a sample of forensic psychiatric patients and found reduced affective modulation of the pupil for a range of negatively valenced stimuli (images and angry faces) and that the reduced affective modulation of the pupil was associated with Factor 1 of the PCL‐R, but not Factor 2. Similarly, Gillespie et al. (2019) found that the pupil dilation caused by emotional faces was reduced in those with high scores on the Meanness scale of the TriPM in a forensic sample, with the deficit being found for all the types of emotional expression that were tested. However, Burley et al. (2017) failed to find any effect of psychopathy on the pupil's reaction to a range of emotional stimuli (images, sounds, and faces) in a community sample. Burley et al. (2020) further tested whether manipulations of attention might reveal an effect of psychopathy on the processing of negative images (for a rational see Newman et al., 2010) but no such effect was found.

The reasons for the difference in the pattern of results between these studies is unclear. First, overall levels of psychopathy are higher in forensic psychiatric patients or prison samples than in the community. Some behavioral or affective correlates of psychopathy may only manifest themselves at high levels of psychopathy and so are not apparent in community samples. These groups (forensic vs community) are also probably different in intelligence, cognitive ability, and/or attentional capacity. Second, gender may also by important. The samples of Burley et al. (2019) and Gillespie et al. (2019) were all male, while that of Burley et al. (2017) was of mixed gender. Gender differences in behavioral correlates of psychopathy have been reported on several occasions (e.g., Vitale & Newman, 2001). It is possible that the addition of female participants in the Burley et al. (2017) community sample had weakened the effect of psychopathy on the affective modulation of the pupil. However, it is notable that Burley et al. (2020) did not find any effects of psychopathy on the affective modulation of the pupil despite using an all‐male sample. Third, different studies have used different models of psychopathy. The study of Burley et al. (2019) used the PCL‐R model of psychopathy with the assessment being completed by professionals. The research of Burley et al. (2017) and Gillespie et al. used the triarchic model of psychopathy and was based on self‐report. These models differ in their conceptualization of psychopathy and there is no simple one‐to‐one correspondence between their scales. Fourth, the different studies have used a variety of stimuli to elicit the affective modulation of the pupil, including affective images (Burley et al., 2017, 2019, 2020), affective sounds (Burley et al., 2017), and emotional expressions on faces (Burley et al., 2017; Gillespie et al., 2019). It is currently unclear how these different stimuli‐types may lead to differences in affective modulation of the startle response in relation to psychopathy.

## 2 PSYCHOPATHY AND PERSONALITY

Psychopathy is a form of personality disorder, and therefore theories of psychopathy need to link closely to theories of personality in general (Miller & Lynam, 2015). Indeed, linking research using measures of psychopathy to measures of general personality has been recommended (Lilienfeld, 2018). Cloninger's psychobiological model (Cloninger et al., 1993) has known relationships to different personality disorders (Svrakic et al., 1993) and has been examined in relationship to psychopathy (Basoglu et al., 2011; Lennox & Dolan, 2014; Martínez‐López et al., 2019; Mikaeili et al., 2020; Snowden & Gray, 2010) as well as to related antisocial/aggressive behaviors (Falk et al., 2017; Miller & Lynam, 2001).

Four of the seven personality traits described by Cloninger et al. (1993) appear to be related to psychopathy. First, Novelty Seeking describes a wish for thrills and adventure, a lack of tolerance for monotony, and a tendency to act quickly or impulsively. It has been positively related to psychopathy in all studies, but more strongly to Factor 2 than Factor 1 (Lennox & Dolan, 2014; Snowden & Gray, 2010). Second, people low on Reward Dependence are described as practical and cold, are rather insensitive to the feelings of others, and make little effort to please others. Psychopathy has been found to be negatively related to Reward Dependence with Facet 2—Affective (Snowden & Gray, 2010) or Facet 3—Lifestyle (Lennox & Dolan, 2014) being the most associated. Thirdly, Cooperativity describes aspects of tolerance, empathy, and being principled. All studies have found that personality factor is negatively related to psychopathy and to most of the subscales, save for Facet 1 (Interpersonal) (Lennox & Dolan, 2014; Snowden & Gray, 2010). Finally, the scale of Harm Avoidance measures a constellation of excessive worrying, pessimism, fearfulness, and shyness. Most studies show a strong negative relationship of this scale to psychopathy, and particularly to the Interpersonal (Facet 1) scale (Snowden & Gray, 2010). However, the study of Basoglu et al. (2011) reports strong *positive* relationships to both Factor 1 and Factor 2 of the PCL‐R. The reason for these very different and opposite results is unknown.

## 3 AIMS AND HYPOTHESES

The study aimed to examine which aspects of psychopathy were associated with a reduction in the emotional processing of stimuli. To this end we operationalized these variables in the following manner:

### 3.1 Measurement of psychopathy

Given the differences in models of psychopathy provided by the PCL‐R conceptualization and the triarchic conceptualization it seemed prudent to measure both models. The SRP‐4 (Paulhus et al., 2016) is based on the PCL‐R model and the TriPM (Patrick, 2010) is based on the triarchic model. Both instruments are self‐report questionnaires designed for use in community samples. We also included a questionnaire designed to measure general personality traits (the TCI; Cloninger et al., 1994) to examine if these normal personality traits, some of which have been shown to be raised in those with psychopathy (see above), were also related to alterations in affective processing.

### 3.2 Index of emotional processing

We indexed emotional processing by the extent of the dilation of the pupil response. While there are several possible psychophysiological methods available (e.g., skin conductance, heart rate variability, blood pressure, modulation of the startle response, etc.) the pupillometry was chosen due to its relative novelty in the field and therefore its ability to provide new insights to complement other techniques (see Burley et al. (2017) for further discussion of the use of pupillometry).

### 3.3 Affective stimuli

We chose to use affective sounds (rather than images) as we felt that these might have the ability to induce greater emotional response than images, and do not induce a pupillary light reflex that can interfere with the isolation of the dilation component of the pupil, which is indicative of the processing of the affective component of the stimulus (see Bradley et al., 2017; Snowden et al., 2016). Affective sounds are known to produce pupil dilation (Burley et al., 2017; Partala & Surakka, 2003) and the processing of the affective component of sounds has been suggested to be compromised in those with high levels of psychopathy when indexed via skin conductance responses (Verona et al., 2004).

Based on previous work (see review above) we made the following hypotheses:

1. Hypothesis 1. Affective sounds would produce greater pupil dilation than neutral sounds.
2. Hypothesis 2. Higher rates of total psychopathy would be related to a reduction in the pupil's response to unpleasant sounds.
3. Hypothesis 3. This reduction would be related to the traits of psychopathy pertaining to callous and affective components of psychopathy (see Gillespie et al., 2019). Specifically, Facet 2‐Affective scale of the SRP‐4, and the Meanness scale of the TriPM.
4. Hypothesis 4. Relatedly, we hypothesized that the Reward Dependence and Cooperativity scales of the TCI would also be associated with an increase in pupil response given their negative association with the callous and affective component of psychopathy.

While our hypotheses concerned the total psychopathy score, specific subscales of the personality questionnaires, and the processing of the unpleasant sounds, we also examined the other relationships (e.g., to the pleasant sounds). These analyses were regarded as exploratory.

## 4 METHOD

### 4.1 Participants

An a priori power analysis based on a small to medium effect size (*r* = 0.25) with *α* = 0.05 (one‐tailed) and 1‐*β* = 0.80 indicated a sample size of 98 participants. However, we oversampled to account for loss of data that typically occurs in pupillometry.

One hundred and twenty‐five participants were recruited into the study via advertisement and word of mouth. All were men aged between 18 and 45 (M = 23.53, SD = 5.20). The participants were a combination of students and members of the public who in return for taking part in the study were entered into a raffle to win a rugby shirt signed by a famous local rugby player. All were required to have normal to corrected vision, self‐reported good hearing, and to be fluent in English. Furthermore, participants were asked to write down any medication they were taking as certain medications can affect pupil dilation. Every participant gave written informed consent. Ethical approval for the study was given by the Ethics Committee of the Department of Psychology, Swansea University.

### 4.2 Materials and design

Thirty sound‐clips were selected from the International Affective Digitised Sounds (IADS; Bradley & Lang, 2007) consisting of 10 unpleasant sounds containing a range of negative stimuli such as threats (animals growling) and distress (crash with screams), 10 pleasant sound clips containing a range of positive stimuli such as happiness (people laughing, crowd cheering) and excitement (lift off, screams of excitement) but no erotic stimuli were used, and neutral sound clips (e.g., bird noises, people talking, engine noises, etc).

All affective categories differed significantly in subjective valence (unpleasant = 2.87; pleasant = 71.7; neutral = 5.06; *p*s < 0.001). The arousal ratings (unpleasant = 7.09; pleasant = 6.76; neutral = 5.05) were greater for both the pleasant and unpleasant sounds in comparison to the neutral sounds (*p*s < 0.001) but the arousal ratings for the pleasant and unpleasant sounds did not differ significantly (*p* = 0.15). Sound‐clips did not differ across the three groups for average and maximum root mean square decibel level *(p*s > 0.25).

Sound‐clips were played to participants at a comfortable set volume through headphones (Sennheiser 201). The sound‐clips were presented for 6000 ms and presentation order was randomized. A grey fixation slide (30 cd/m^2^) was displayed throughout. There was an inter‐trial interval of 10,000 ms to allow for the pupil to return to baseline.

### 4.3 Self‐report psychopathy scale V4

The SRP‐4 (Paulhus et al., 2016) consisted of 64 items and each involved a statement about a behavior or attitude one might have about themselves, other people, or the world in general. The 64 items group into four scales (16 items each) of Facet 1—Interpersonal (e.g., “*Most people are wimps*”), Facet 2—Affective (e.g., “*A lot of people are “suckers” and can easily be fooled*”), Facet 3—Lifestyle (e.g., “*I like to have sex with people I barely know*”), and Facet 4—Antisocial (e.g., “*I have broken into a building or vehicle in order to steal something or vandalize*”). Each statement was followed by five‐point Likert scale: 1 = Strongly Disagree, 2 = Disagree, 3 = Neutral, 4 = Agree and 5 = Strongly Agree. Missing responses in the all the questionnaires were addressed by pro‐rating the average for the relevant subscale. However, this was rarely needed as there were <1% incomplete responses. As well as providing the Facet scores, the SRP‐4 also gives a total psychopathy score.

The factor structure of the SRP has been validated by confirmatory factor analysis in a number of samples (Mahmut et al., 2011; Neal & Sellbom, 2012; Paulhus et al., 2016). The scales have also shown to have good reliability in college samples (Total = 0.92; Interpersonal = 0.83; Affective = 0.79; Lifestyle = 0.80; Antisocial = 0.76; (Paulhus et al., 2016)). In the present sample the total score and each of the scales had acceptable reliability (Total = 0.88; Interpersonal = 0.80; Affective = 0.79; Lifestyle = 0.72; Antisocial = 0.70).

### 4.4 TriPM

The Triarchic Psychopathy Measure (TriPM; Patrick, 2010) is a self‐report measure consisting of 58 items. Each item contains a statement about a person (e.g., “*I’m a born leader”)* and the person indicated the degree to which they agreed with the statement on a four‐point scale (*false*, *mostly false*, *mostly true*, *true*) and these responses were used to score the items from 0 to 3. The TriPM has three scales: Boldness (19 items), Meanness (19 items), and Disinhibition (20 items). The TriPM does not provide a method for combining the scale scores into a total psychopathy score. We decided not to simply add the scale scores, as scores on the Boldness scale are typically much higher than for the other two scales (see Table 1 for mean scores in the present sample). Therefore, a total TriPM score was calculated by z‐scoring each of the scales and adding these three z‐scores.

**TABLE 1:** Descriptive statistics for the SRP‐4 and TriPM measures of psychopathy and the zero‐order correlations between the measures

	TriPM scales					
	Total	Boldness	Meanness	Disinhibition	M	SD
SRP4						
Total	**0.82**	**0.34**	**0.72**	**0.63**	157.0	22.3
Interpersonal	**0.68**	**0.39**	**0.60**	**0.41**	42.7	9.0
Affective	**0.60**	0.18	**0.71**	**0.33**	40.7	7.0
Lifestyle	**0.66**	**0.38**	**0.43**	**0.57**	48.1	8.0
Antisocial	**0.34**	−0.03	**0.27**	**0.45**	25.6	7.8
M	68.5	34.3	17.5	16.7		
SD	15.5	7.2	7.8	7.4		

Note

Figures in bold: correlation *p* < 0.01.

The TriPM has been shown to validly measure the construct of psychopathy when related to the PCL‐R (Venables et al., 2014). Good internal consistency and test‐retest reliability have also been demonstrated for the TriPM (Blagov et al., 2016). In the present sample each of the scales had acceptable reliability (Boldness = 0.82; Meanness = 0.86; Disinhibition = 0.84).

### 4.5 TCI

The Temperament and Character Inventory (TCI: Cloninger et al., 1994) is a self‐report questionnaire consisting of 240 questions which required a true or false answer. The answers are summed to form seven scales: Novelty Seeking (e.g., “*I often try new things just for fun or thrills*, *even if most people think it is a waste of time*”), Harm Avoidance (e.g., “*I often feel tense and worried in unfamiliar situations*, *even when others feel there is little to worry about*”), Reward Dependence (e.g., “*I am often moved deeply by a fine speech or poetry”*), Persistence (e.g., “*I could probably accomplish more than I do*, *but I don't see the point in pushing myself harder than is necessary to get by*”), Self‐Directedness (e.g., “*I often wish that I was smarter than everyone else*” reverse‐scored), Cooperativeness (e.g., “*I often consider another person's feelings as much as my own*”), and Self‐transcendence (e.g., “*Often when I am concentrating on something*, *I lose awareness of the passage of time*”).

The factor structure and reliability of the TCI has been demonstrated many times across the world (Cloninger et al., 1994). For instance, Sung et al. (2002) show reliabilities ranging from 0.60 to 0.87 for a sample of Korean college students. Similar figures were obtained in the present sample (*α*s > 0.70) therefore all scales were deemed to have acceptable reliability.

### 4.6 Data acquisition and reduction

A Tobii X2‐60 Hz eye tracker recorded pupil data throughout each task which allowed free movement of the head during the task. Accuracy for binocular tracking was 0.4 degrees and precision was 0.34 degrees. The eye tracker was calibrated to each participant's eyes before commencement of the task using a 5‐point calibration screen. The participants viewed the blank screen of a 39.60 cm laptop display monitor positioned 57 cm from the viewer's eyes. The screen emitted a luminance of 30 cd/m^2^. The angle of the screen was adjusted for each participant such that their eyes were in the center of the calibration window. Data was cleaned and analyzed using Matlab (MathWorks, version 8.5). We removed any pupil diameter increase or decrease of 0.375 mm within one data reading (over a period of approximately 16.67 ms) as these are thought to be an artefact (Partala & Surakka, 2003). We also deleted the first data point that followed missing data to avoid abnormal readings. Pupil size was determined by calculating the mean diameter across both eyes. Initial pupil diameter for each trial was calculated over the period 200 ms prior to stimulus‐onset (Snowden et al., 2016). For every trial, initial pupil diameter was subtracted from subsequent pupil size to establish baseline‐corrected pupil diameter.

Trials were omitted if there was less than 50% data for the selected time‐window. Participant means were only calculated for each affective category if at least 50% of trials held valid data. Participants were excluded if they recorded less than 50% valid data across all trials during stimulus presentation, resulting in differing sample sizes across tasks.

### 4.7 Procedure

Participants attended the experimental laboratory individually. The pupillometry measurements took place in a quiet laboratory with dim lighting. Participants wore headphones that served to avoid any distracting noise that might occur and to deliver the sound clips. After completion of the pupillometry task (which took approximately eight minutes) participants completed the questionnaires in a nearby antechamber to the laboratory (which took approximately 15 min). They were then debriefed and given course credit or payment for their time.

### 4.8 Data analytic plan

**FIGURE 1:**
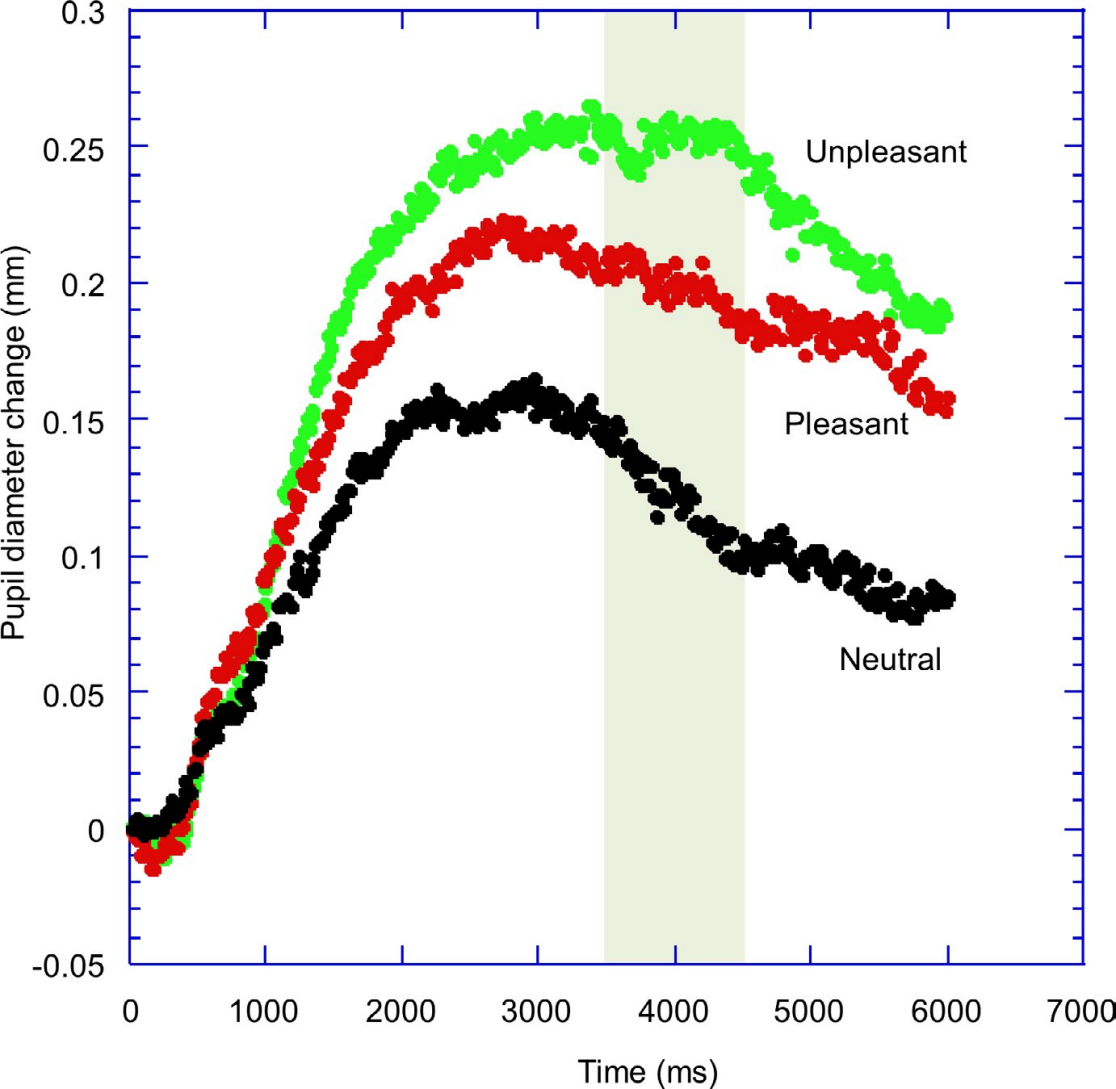
The change in pupil size (relative to pre‐stimulus) is plotted as a function of time for the three types of sound (neutral, pleasant, unpleasant)

It was expected that both categories of affective stimuli (pleasant and unpleasant sounds) would produce greater pupil dilation than the neutral stimuli (see Figure 1 for the actual results). In order to quantify these effects, we took a time window in the region where there was strong differentiation between the neutral and affective stimuli (3500–4500 post stimulus onset) and calculated the average pupil diameter in this period. Our main hypotheses were that the magnitude of modulation due to the affective component of the stimuli would be less for those with high psychopathic traits related to the affective components of psychopathy. To test this hypothesis, we calculated an emotional index for each affective category separately by subtracting the response to the neutral stimulus from the affective stimulus. We then calculated the zero‐order correlations between the psychopathy subscales and the affective modulation of the pupil for each measure of psychopathy and general personality as indexed by the TCI. The facets of the SRP‐4 typically show quite substantial correlations and so we also performed linear regression analyses separately for the four‐facet version of the SRP‐4 model and report their beta‐weights. A similar regression was also performed for the TriPM scales and for the TCI scales.

## 5 RESULTS

Six participants were removed due to excessive missing data. Data from the measures of psychopathy and from the pupil responses were inspected for outliers (>3 SD) and one participant was removed. For the remaining 118 participants, levels of skew and kurtosis were small (<1.0) and well within acceptable ranges for all scales. Visual inspection confirmed that all data appeared to conform well to normal distributions and so parametric statistics were used for all analyses.

### 5.1 Personality and psychopathy scales

Table 1 gives the descriptive statistics and correlations for two measures of psychopathy. For the SRP‐4, the scores in this sample were similar (though a little higher) that those reported for a mixed‐gender college sample from North America (Paulhus et al., 2016) which may be explained by the present sample being all male. In comparison to an all‐male North American offender sample (Paulhus et al., 2016), the present sample had lower SRP‐4 scores (e.g., 189.7 vs 157.1 for the total score) which the Antisocial scale showing the greatest difference (47.7 vs 25.5). For the TriPM the scores appear similar to those from a North American sample of college students (Donnellan & Burt, 2016). In comparison to an offender sample (Stanley et al., 2013), the present sample showed similar (if not slightly higher) scores on Boldness (31.7 vs 34.3), but lower scores on meanness (22.5 vs 17.4) and disinhibition (33.7 vs 16.7).

The two measures of psychopathy showed good agreement at the total level score (*r* = 0.82). However, at the subscale level there is clearly no one‐to‐one relationship between the scales from each instrument. For example, TriPM disinhibition appears to be correlated moderately with all the four facets of the SRP‐4. However, for the purposes of the present research, it is noteworthy that TriPM Meanness showed the expected strong correlation with Facet 2‐Affective of the SRP‐4.

Table 2 presents the correlations between the TCI and the measures of psychopathy. As the aim of this research was to examine the relationship between measures of psychopathy/personality to affective processing as indexed by the affective modulation of the pupil, only a few points will be noted here. First, as predicted, Reward Dependence was negatively correlated with most aspects of psychopathy, but most strongly with Facet 2—Affective of the SRP‐4 and Meanness of the TriPM (*r*s > 0.50). Cooperativeness was also negatively correlated with most aspects of psychopathy, but most strongly with Facet 2‐Affective and Meanness (*r*s > 0.50). Harm Avoidance was negatively correlated with certain aspects of psychopathy, but most strongly with Boldness (*r* = −0.75). Novelty Seeking was positively correlated with most aspects of psychopathy, but most strongly with Facet 3‐Lifestyle (*r* = 0.58).

**TABLE 2:** Correlations (Pearson's *r*) between the measures of psychopathy (SRP‐4 and TriPM) and the temperament and character inventory (TCI)

TCI	Novelty seeking	Harm avoidance	Reward dependence	Persistence	Self‐directedness	Cooperativity	Self‐transcendence
SRP‐4							
Total	**0.44**	−0.25	**−0.47**	0.08	−0.16	**−0.52**	0.07
Interpersonal	**0.32**	−0.25	**−0.42**	0.15	−0.22	**−0.43**	−0.08
Affective	0.07	−0.07	**−0.66**	0.14	−0.16	**−0.57**	−0.09
Lifestyle	**0.58**	**−0.36**	−0.29	−0.11	−0.03	−0.19	0.09
Antisocial	0.23	0.01	0.05	0.06	−0.04	−0.28	0.27
TriPM							
Total	**0.54**	**−0.37**	**−0.47**	0.05	−0.12	**−0.47**	0.00
Boldness	**0.40**	**−0.75**	−0.17	0.19	**0.36**	−0.01	−0.04
Meanness	0.29	−0.02	**−0.58**	0.00	−0.26	**−0.65**	−0.16
Disinhibition	**0.42**	0.02	−0.21	−0.08	**−0.34**	−0.29	0.20
M (SD)	22.4 (6.6)	12.2 (7.4)	14.1 (4.1)	4.9 (2.1)	27.8 (7.1)	32.1 (6.5)	12.0 (6.0)

Note

Bold figures *r* > 0.30; *p* < 0.001.

### 5.2 Impact of affective sounds

Figure 1 illustrates the averaged pupil response to the sound stimuli. The pupil begins to dilate to the presentation of the sounds with a latency of around 400 ms. This dilation continues with time and reaches maximum levels at around 2500–3000 ms post‐stimulus presentation before a gradual decline. Responses to the affective stimuli appear to be greater than to the neutral stimuli, with the differentiation first occurring at around 1000 ms. To quantify this, we defined a response window where there appears to be greatest differentiation between the affective and neutral stimuli (3500–4500 ms). Average pupil diameter within each of these time windows was calculated for each participant.

Planned analyses revealed that the pupil was larger to unpleasant sounds (0.21 mm) than to neutral sounds (0.14 mm), *t*(117) = 5.31, *p* < 0.001, *d* = 0.45; 95% CI [0.27, 0.63], and to pleasant sounds than to neutral sounds (0.20 mm), *t*(117) = 3.86, *p* < 0.001, *d* = 0.39; 95% CI [0.19, 0.60]. The response to the pleasant and unpleasant sounds did not differ significantly, *t*(117) = 0.93, *p* = 0.34. Hence, Hypothesis 1 was confirmed.

### 5.3 Psychopathy and affective modulation of the pupil response

To quantify the effect of the emotional content of sounds, the response to the neutral stimuli was subtracted from each of the affective sound categories (negative and positive). Table 3 illustrates the correlations between the personality/psychopathy scores and the affective modulation of the pupil's response.

**TABLE 3:** Zero‐order correlations (*r*) and regression weights (*β*) between SRP‐4, TriPM, and TCI scores and pupil diameter in response to negative and positive images (minus pupil diameter to neutral images)

	SRP‐4				
	Total	Interpersonal	Affective	Lifestyle	Antisocial
Negative					
*r*	−0.12	−0.12	−0.23**	−0.02	0.03
*β*		−0.03	−0.23*	0.04	0.07
Positive					
*r*	0.05	−0.03	−0.03	0.12	0.07
*β*		−0.08	−0.03	0.15	0.05

	TriPM			
	Total	Boldness	Meanness	Disinhibition
Negative				
*r*	−0.18*	−0.06	−0.21*	−0.11
*β*		−0.04	−0.19*	−0.02
Positive				
*r*	0.05	−0.01	0.09	0.02
*β*		−0.02	0.11	−0.03

	TCI						
	NS	HA	RD	P	SD	C	ST
Negative							
*r*	−0.05	−0.03	0.19*	−0.01	0.01	0.27**	−0.08
*β*	−0.05	−0.09	0.14	0.04	−0.11	0.24*	−0.14
Positive							
*r*	0.02	−0.02	−0.01	−0.10	−0.06	0.06	−0.03
*β*	0.02	−0.04	−0.03	−0.07	−0.07	0.09	−0.03

Note

*p < 0.05, **p < 0.01.

### 5.4 SRP‐4

To test Hypothesis 2, total SRP‐4 score was correlated with the EI for the unpleasant sounds. However, though in the predicted direction, this correlation was not significant and so Hypothesis 2 was not supported. The four‐facets of the SRP‐4 were then examined in turn. The affective facet (Facet 2) was significantly negatively correlated with the EI for the unpleasant sounds, and its regression weight when all four facets were examined together was also significantly related to the EI score. Hence, Hypothesis 3 was supported. The exploratory analyses of the other facets of the SRP‐4 and all those involving the EI for the positive sounds did not reveal any significant effects.

### 5.5 TriPM

To test Hypothesis 2, total TriPM score was correlated with the EI for the unpleasant sounds. The correlation was negative and significant and hence Hypothesis 2 was supported (though the effect size is “small” by standard conventions). The three scales of the TriPM ‐ were then examined in turn. Meanness was significantly negatively correlated with the EI for the unpleasant sounds, and its regression weight when all three scales were examined together was also significantly related to the EI score.^2^ Hence, Hypothesis 3 was supported. The exploratory analyses of the other TriPM scales and all those involving the EI for the positive sounds did not reveal any significant effects.

### 5.6 TCI

Hypothesis 4 was tested by examining the relationship between the Reward Dependence and the Cooperative scales of the TCI to the EI for the unpleasant sounds. There was a positive association between the affective modulation of the pupil for the negative stimuli for both the Reward Dependence and Cooperativeness scales, although the former was not significant for the regression analysis. Hence, Hypothesis 4 was supported. No other associations were significant.

## 6 DISCUSSION

The present study examined the hypothesis that psychopathy, and personality traits associated with psychopathy as indexed by the TCI, is associated with reduced pupil dilation in response to affective auditory stimuli. We hypothesized that the affective components of psychopathy would be related to reduced affective modulation of the pupil response. The hypothesis was supported by showing that the Affective scale of the SRP‐4, and the Meanness scale of the TriPM were negatively correlated to pupil dilation to the unpleasant sounds.

In the present study, the association between the Affective scale of the SRP‐4 and the Meanness scale of the TriPM was very high (*r* > 0.70). It is, therefore, not surprising that these two scales gave similar results in relation to the affective modulation of the pupil. The Affective scale and the Meanness scale both describe individuals with a lack of empathy, and a tendency to social aggression. In turn, both these scales of psychopathy were strongly (negatively; *r*s > 0.57) associated with Reward Dependence and Cooperativeness on the TCI. Hence, these two scales were also associated with affective modulation of the pupil.

### 6.1 Pleasant vs unpleasant stimuli

It is notable that all the significant results with respect to psychopathy were for the unpleasant stimuli. There has been considerable debate on whether the emotional dysfunction found in psychopathy is general to all emotions, specific to negative emotions, or specific to a single emotion such as fear. For example, Esteller et al. (2016) found that the fear potentiated startle dysfunction due to the Boldness scale of the TriPM was specific to threat stimuli, with the startle response to other unpleasant stimuli (such as mutilations) and pleasant stimuli (erotic images) not being related to any aspect of psychopathy. The present results give support to the notion that the psychopathic emotional dysfunction, at least that relating to the affective/meanness component of psychopathy, is limited to unpleasant sounds. However, the unpleasant stimuli we used were a mixture of negative emotions (for example, some were screams and some were people crying) and it may be useful in future research to examine specific negative emotions in turn.

It is also noticeable (see Figure 1) that the dilation produced by the pleasant sounds was not as great as that produced by the unpleasant sounds (though this was not statistically significant). Hence, it is possible that the differential effects of psychopathy/general personality for the pleasant and unpleasant sounds might be due to the greater effect of the unpleasant sounds (perhaps due to greater arousal), rather than a differential effect of valence per se. Further studies are needed to differentiate these possibilities.

### 6.2 Relationship to other studies of affective modulation of the pupil

Previous studies of the relationship between affective modulation of the pupil and psychopathy have produced somewhat mixed results. For instance, Burley et al. (2017) using similar auditory stimuli did not find any relationship between affective modulation of the pupil and TriPM psychopathy in a mixed‐gender sample. The effects found in the current study are “small” and it is not difficult to see that they could easily be missed in studies with smaller sample sizes, or in samples with mixed gender if the effects are gender‐specific to males. Thus, at present, while our results are evidence of a reduced processing of emotional material for people with high levels of the affective/meanness traits of psychopathy, further work is needed to better understand under what conditions these effects occur. For instance, other studies have shown that the levels of attention to the emotional stimuli (Dvorak‐Bertsch et al., 2009) or the “complexity” of the emotional stimuli (Sadeh & Verona, 2012) can moderate the effects of psychopathy on the processing of affect‐laden images or sounds.

The studies of by Burley et al. (2019) and Gillespie et al. (2019) have shown reduced processing of emotional material for people with high traits of psychopathy which were related to both interpersonal and affective traits (Burley et al., 2019) and to affective traits (Gillespie et al., 2019). As such the current findings are supportive of the notion that it is these affective/meanness traits that are related to reduced emotional processing in psychopathic individuals.

We believe that this is the first study to present physiological evidence related to emotional processing deficits related to Cloninger's psychobiological model of personality (Cloninger et al., 1993). In particular, the scales of Reward Dependence and Cooperativity were related to reduced processing of negative affective information. The Reward Dependence scale measures a person's tendency to respond markedly to signals of interpersonal reward, particularly those of social approval, support, and sentiment. Hence, individuals low in Reward Dependence are tough‐minded, socially detached, and insensitive to social cues (Cloninger et al., 1993). This description fits well with the present findings that those loading low on this scale are relatively immune to the negative affective information of the sounds presented. However, from this description we would have also expected reduced processing of positive information (but see earlier discussion).

Cooperativeness refers to the degree a person is agreeable in their relationship with others. Hence, individuals low in cooperativeness are thought to be callous, aggressively self‐centered, and hostile (Cloninger et al., 1993). The present study shows these traits are highly correlated with the Affective scale of the SRP‐4, and the Meanness scale of the TriPM. It appears that such individuals show reduced affective processing of the unpleasant sounds (or that this information does not cause the same emotional reaction). This blunting of affect may then cause the person to appear to be insensitive and uncaring about the feelings of others.

### 6.3 Limitations

The study used an all‐male community sample, including university students. It is likely that levels of psychopathy are far lower in this community sample than in forensic or psychiatric settings. There are some advantages to using community samples, where problems such as poor literacy, poor functioning due to substance use or other problems associated with a criminal lifestyle, should be reduced. However, it is possible that many psychopathic deficits may not be manifest until high levels of psychopathy are reached (Zimak et al., 2014), or that these deficits are masked or compensated for by strategies dependent on good intelligence or emotional resilience (Gao & Raine, 2010; Ishikawa et al., 2001). Clearly, there is a need for these findings to be expanded into other populations with higher levels of psychopathy and criminality, and to female samples before our results could be generalized (see Kimonis et al., 2020 for a further discussion of these issues).

Our main hypothesis (Hypothesis 3) was that the Affective scale of the SRP‐4, and the Meanness scale of the TriPM that would be related to emotional deficits for unpleasant sounds. As such we did not correct our alpha level for these tests. We also performed multiple exploratory analyses on the other scales and image types without correcting our alpha level. We felt this justified as these analyses were exploratory. However, none of these analyses produced significant results even for this alpha level.

Further, while the crucial effects may be significant, they are small in terms of effect size. Further work is needed to improve this paradigm to produce a more sensitive assay of emotional processing. One promising line of research might be to use sounds that elicit more specific negative emotions, such as distress, or fear, or threat, rather than just “negative” emotions per se (Libkuman et al., 2007; Mikels et al., 2005). It is clear that all negative‐valenced stimuli (and positively‐valenced stimuli) do not produce similar results even when levels of valence and arousal are well matched (e.g., Carretié et al., 2011; Van Hooff et al., 2013) and these differences may lead to differential effects in those with psychopathy (see Levenston et al., 2000).

The present study has focused on the pupillometry as the measure of emotional processing and did not take other psychophysiological measures (e.g., skin conductance, heart rate, startle reflex), nor any self‐report or behavioral measures of emotional processing. Examining the relationship between these different assays of emotional processing is clearly of great importance given the different pattern of results found between studies. For example, Estellar et al. (2016) show that the Boldness component of psychopathy is related to reduced startle response to threat stimuli, whereas the present study shows it is the Meanness component that is related to reduced pupil dilation to unpleasant sounds. Studies where these different assays are taken in the same participants to the same stimuli at the same time would provide a more powerful study of these components of emotional processing and how they are affected in those with high psychopathic traits.

## 7 CONCLUSIONS

In conclusion, using changes in pupil size in response to stimuli with affective content, the current data supports the hypothesis that the affective traits of psychopathy, and general personality traits indexed by the TCI that are related to these affective psychopathic traits, are associated with a deficit in processing negatively valenced affective information, whereas no such deficit was found for the other traits of psychopathy. The current pupillometry paradigm has obvious practical application as a fast, relatively cheap, and non‐intrusive measure to identify individuals with problems in processing negative affective stimuli who may benefit from interventions targeting these core affective impairments.

## CONFLICT OF INTEREST

The Authors declare that there is no conflict of interest.

## ETHICAL APPROVAL

This study was obtained from the Department of Psychology Ethics Committee, Swansea University.

## AUTHOR CONTRIBUTION

Nicola Gray: Conceptualization, Methodology, Supervision, Writing‐ Reviewing and Editing. Aimee McKinnon: Software, Investigation, Validation. Robert J. Snowden: Conceptualization, Formal analysis, Visualization, Writing—Original Draft.

## Footnotes

^1^
Though see Anderson et al. (2011) for a failure to replicate even this finding.

^2^
Some previous studies have suggested that emotional dysfunction in psychopathy is not related to any subscale of psychopathy but is related to their interaction(s). For instance, Kimonis et al. (2020) show that reduced attention to negative images is related to increases in Boldness and increase Meanness only when Disinhibition scores were high. On the other hand, Maes and Brazil (2015) showed that distraction due to emotional images was reduced in those with fearlessness traits only if impulsive‐antisocial traits were low. We therefore tested these interaction models by adding the interaction term(s) to the regression model. None of the interaction terms increased the model's fit, nor were any of the interaction terms significant.
